# Analgesic and Antipyretic Activities of Methanol Extract and Its Fraction from the Root of* Schoenoplectus grossus*

**DOI:** 10.1155/2016/3820704

**Published:** 2016-02-11

**Authors:** Nirmal Kumar Subedi, S. M. Abdur Rahman, Mohammad Ahsanul Akbar

**Affiliations:** ^1^Faculty of Pharmacy, Little Buddha College of Health Science, Purbanchal University, Kathmandu 44600, Nepal; ^2^Department of Clinical Pharmacy & Pharmacology, Faculty of Pharmacy, University of Dhaka, Dhaka 1000, Bangladesh

## Abstract

The study aims to evaluate analgesic and antipyretic activities of the methanol extract and its different fractions from root of* Schoenoplectus grossus* using acetic acid induced writhing and radiant heat tail flick method of pain models in mice and yeast induced pyrexia in rats at the doses of 400 and 200 mg/kg. In acetic acid writhing test, the methanol extract, petroleum ether, and carbon tetrachloride fractions produced significant (*P* < 0.001 and *P* < 0.05) inhibition of writhing responses in dose dependent manner. The methanol extract at 400 and 200 mg/kg being more protective with 54% and 45.45% of inhibition compared to diclofenac sodium of 56% followed by petroleum ether fractions of 49.69% and 39.39% at the same doses. The extracts did not produce any significant antinociceptive activity in tail flick test except standard morphine. When studied on yeast induced pyrexia, methanol and petroleum ether fractions significantly lowered the rectal temperature time dependently in a manner similar to standard drug paracetamol and distinctly more significant (*P* < 0.001) after second hour. These findings suggest that the root extracts of* S. grossus* possess significant peripherally acting analgesic potential and antipyretic property. The phytochemical screening showed the presence of flavonoids, alkaloids, and tannins.

## 1. Introduction

Various injuries and diseases are most often presented with pain and fever. Nonsteroidal anti-inflammatory drugs (NSAIDs) are commonly prescribed drugs for their management but significant gastrointestinal complications like perforation, bleeding, peptic ulcers, and obstructions have limited their uses in clinical settings [1, 2]. Selective COX-2 inhibitors have some benefits on lowering such side effects while risk of cardiovascular adverse events demands important consideration [3–6]. The social abuse and other side effects like psychological dependency, addiction, tolerance, sedation, constipation, and respiratory depression associated with narcotic analgesics are playing negative role in management of chronic pain and sometimes being inadequate [7]. The negative consequences of pain are even broader with huge economic and social burden on individual and society affecting overall quality of life and working status [8]. Moreover, currently available pain relieving drugs are not so effective in subsiding pain, only contributing to 50% relief in about 30% of the patients in some cases which suggest dire need for effective analgesics [9]. With these sorts of shortcomings and other associated problems with analgesics, search for newer drugs to treat pain and fever is going to be very essential. Alternative medicines from natural sources are important options in this regard as around 25% of all currently available synthetic medicines are directly or indirectly based on medicinal plants [10]. Lower side effects, wide distribution, and traditional uses of medicinal plants provide suitable sources for the development of new drugs.

*Schoenoplectus grossus* (syn.* Actinoscirpus grossus*) commonly known as Kesur in Bengali language belongs to family Cyperaceae. It is commonly found in fresh-water swamps, edges of ponds, and moist places. This plant is native in Bangladesh, Nepal, India, and other Southeast Asian countries. The root of this plant is used traditionally as diuretic and against various infections, burning sensitive, gonorrhea, and fever [11]. Since this plant has not been explored for such activity, the present study was designed to evaluate analgesic activity of methanol extract and its fractions from root of* Schoenoplectus grossus* (*S. grossus*) using two animal models of pain in mice, acetic acid induced writhing and radiant heat tail flick method, and at the same time antipyretic activity against yeast induced pyrexia in rats.

## 2. Materials and Methods

### 2.1. Plant Materials

The roots of plant* Schoenoplectus grossus* were collected from Barisal area, Bangladesh. The plant was authenticated by an expert taxonomist and a specimen representing this collection has been deposited in the Dhaka University Herbarium, Dhaka, for further reference (accession number DUSH1247).

### 2.2. Extraction of Plant Material

The roots of the plant were sun dried for several days and ground into coarse powder using high capacity grinding machine. The powdered material (600 gm) was taken in separate clean, round bottomed flask and soaked in 2.5 mL of methanol. The container with its content was sealed by cotton plug and aluminum foil and kept for a period of 15 days accompanying occasional shaking and stirring. The whole mixture was then filtered through cotton followed by Whatman number 1 filter paper. The filtrate obtained was concentrated at 39°C with a Heidolph rotary evaporation at low temperature and pressure to obtain methanol extract. The (crude) methanol extract was then air dried to solid residue and kept in standard condition. The methanol extract was then partitioned by different solvents petroleum ether, dichloromethane, ethyl acetate, and carbon tetrachloride by Modified Kupchan Partition Method [12]. The partitioned fractions were then air dried to solid residue and kept for further study.

### 2.3. Chemicals

Paracetamol and diclofenac sodium used were obtained from ACI Pharmaceuticals Ltd.; morphine and yeast were purchased from Gonoshasthaya Pharmaceuticals Ltd., Dhaka, Bangladesh. Acetic acid, DMSO, and Tween-80 were of Merck Chemicals Ltd., Germany, while sterile normal saline (0.9% Nacl, Beximco Infusions Ltd., Bangladesh) was used for the study. All solvents and chemicals used were of analytical grade standard. All solutions were prepared on the same day of experiments.

### 2.4. Experimental Animal

Albino Wistar rats (120–150 g) and Swiss albino mice (25–30 g) of either sex, purchased from the animal resource branch of Jahangirnagar University, Dhaka, were used for the study. The animals were housed under standard environmental conditions at room temperature, humidity (50 ± 5%), and 12-h light-dark cycles in sanitized polypropylene cages containing sterile paddy husk as bedding. They had free access to standard pellets as basal diet and water* ad libitum*. All the animals were acclimatized for seven days before the study. The animals were randomized into experimental and control groups and water was given* ad libitum* but food was withdrawn 12 hours before and during the experimental hours. All experimental protocols were in compliance with Dhaka University Ethics Committee on Research in Animals as well as internationally accepted principles for laboratory animal use and care. The ethics for use of experimental animals were followed strictly.

### 2.5. Phytochemical Screening

The preliminary phytochemical screening of methanol root extract and its different fractions were performed using standard protocols [13].

### 2.6. Analgesic Activity

#### 2.6.1. Acetic Acid Induced Writhing Test

The method described by Koster et al. was used for the evaluation of analgesic activity in mice [14]. The experimental animals were weighed and randomly divided into 10 groups consisting of 5 mice in each. Group I (control) received 1% Tween-80 in normal saline (10 mL/kg) orally. Group II (positive control) received standard drug diclofenac sodium at oral dose of 50 mg/kg. Remaining groups were treated orally as follows:

 Groups IIIA and IIIB: they received methanol extract at doses of 400 and 200 mg/kg.
 Groups IVA and IVB: they received petroleum ether fraction at doses of 400 and 200 mg/kg.
 Groups VA and VB: they received carbon tetrachloride fraction at doses of 400 and 200 mg/kg.
 Groups VIA and VIB: they received dichloromethane fraction at doses of 400 and 200 mg/kg.
 Groups VIIA and VIIB: they received ethyl acetate fraction at doses of 400 and 200 mg/kg.

All treatments were administered orally. 45 minutes after administration of standard drug and test samples, each mouse was injected with 0.7% acetic acid at the dose of 10 mL/kg body weight intraperitoneally. The number of writhing responses produced by each mouse was recorded for 15 minutes commencing just 5 minutes after acetic acid injection. The percentage of analgesic activity was calculated as follows:

(1) %  inhibition  of  abdominal  writhing=Wc−WtWc×100,

where *W* is number of writhings, *c* is control, and *t* is test.

#### 2.6.2. Radiant Heat Tail Flick Test

The analgesic activity against thermal stimuli was evaluated using method described by D'amour and Smith [15] in mice with minor modifications in the procedure. A radiant heat tail flick Analgesiometer (Medicraft, India) was used to measure the reaction latencies. The radiant heat source (55°C ± 2) was maintained and the distal part of tail at 2.5 cm measured from the root of the tail was placed in the source to record the reaction time and the time taken by mice to remove its tail was taken as end point. The experimental animals were weighed and randomly divided into 10 groups consisting of 5 mice in each. Group I (control) received 1% Tween-80 in normal saline (10 mL/kg) orally. Group II (positive control) was treated with standard drug morphine sulphate (2 mg/kg) subcutaneously. Remaining groups were treated orally as follows:

 Groups IIIA and IIIB: they received methanol extract at doses of 400 and 200 mg/kg.
 Groups IVA and IVB: they received petroleum ether fraction at doses of 400 and 200 mg/kg.
 Groups VA and VB: they received carbon tetrachloride fraction at doses of 400 and 200 mg/kg.
 Groups VIA and VIB: they received dichloromethane fraction at doses of 400 and 200 mg/kg.
 Groups VIIA and VIIB: they received ethyl acetate fraction at doses of 400 and 200 mg/kg.

The latent period of the tail flick response was determined at 30, 60, and 90 minutes after the administration of drugs and test samples. In order to avoid the skin damage, the cut-off reaction time was kept at 16 sec. The percentage elongation of reaction time was then calculated to evaluate antinociceptive activity.

### 2.7. Antipyretic Activity

#### 2.7.1. Yeast Induced Hyperthermia in Rats

The antipyretic activity was evaluated with fever induced by Brewer's yeast following the established method [16, 17] in rats with some modifications. At zero hour, the basal rectal temperature of each rat was recorded using clinical digital thermometer. Pyrexia was induced by subcutaneous injection of 15% w/v suspension of Brewer's yeast in distilled water at a dose of 10 mL/kg body weight. After 18 hr of Brewer's yeast injection the rise in rectal temperature was recorded and only animals showing an increase in temperature of at least 0.6°C (or 1°F) were selected for the study. The animals were randomly divided into four groups, each group containing five rats. Group I received 1% Tween-80 in normal saline orally. Group II was given standard drug paracetamol at the dose of 100 mg/kg perorally. Groups IIIA and IIIB received methanol extract at oral dose of 400 mg/kg and 200 mg/kg. Groups IVA and IVB received petroleum ether extract at dose of 400 mg/kg and 200 mg/kg orally. After the treatment, the temperature of all the rats in each group was recorded at 0 hr, 1 hr, 2 hr, 3 hr, and 4 hr.

### 2.8. Statistical Analysis

All values were expressed as the mean ± standard error of the mean (SEM) and the results were analyzed statistically by one-way analysis of variance (ANOVA) for analgesic activity and multivariate analysis of variance (MANOVA) for antipyretic effect through time followed by Dunnett's post hoc multiple comparison test by using SPSS ver. 16. For MANOVA, Levene's test of equality errors of variance was performed. *P* < 0.05 was considered to be statistically significant.

## 3. Result

### 3.1. Phytochemical Screening

The phytochemical analysis of the methanol root extract from* Schoenoplectus grossus* as well as petroleum ether fractions has shown the presence of alkaloids, tannins, and flavonoids whereas phytoconstituents of other fractions are depicted in Table 1.

### 3.2. Acetic Acid Induced Writhing Test

**Figure 1:**
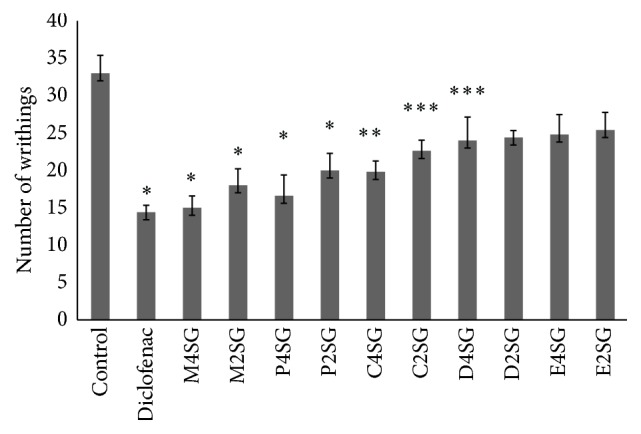
Analgesic activity of standard diclofenac, M4SG: methanol extract 400 mg/kg, M2SG: methanol extract 200 mg/kg, P4SG: petroleum ether fraction 400 mg/kg, P2SG: petroleum ether 200 mg/kg, C4SG: carbon tetrachloride fraction 400 mg/kg, C2SG: carbon tetrachloride 200 mg/kg, D4SG: dichloromethane fraction 400 mg/kg, D2SG: dichloromethane 200 mg/kg, E4SG: ethyl acetate fraction 400 mg/kg, and E2SG: ethyl acetate 200 mg/kg of* S. grossus* in acetic acid writhing. Values are represented in mean ± SEM (*n* = 5). One-way ANNOVA followed by Dunnett's multiple comparisons was performed. *P* < 0.05 was considered significant compared to control, ^*∗*^ *P* < 0.001, ^*∗∗*^ *P* < 0.005, and ^*∗∗∗*^ *P* < 0.05.

**Figure 2:**
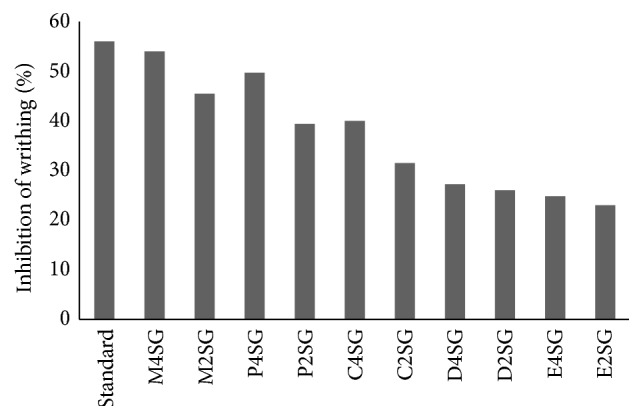
Percentage inhibition by standard: diclofenac sodium, M4SG: methanol extract 400 mg/kg, M2SG: methanol extract 200 mg/kg, P4SG: petroleum ether fraction 400 mg/kg, P2SG: petroleum ether 200 mg/kg, C4SG: carbon tetrachloride fraction 400 mg/kg, C2SG: carbon tetrachloride 200 mg/kg, D4SG: dichloromethane fraction 400 mg/kg, D2SG: dichloromethane 200 mg/kg, E4SG: ethyl acetate fraction 400 mg/kg, and E2SG: ethyl acetate 200 mg/kg of* S. grossus* in acetic acid writhing.

The effect of methanol extract and its fraction of* Schoenoplectus grossus* on acetic acid induced writhing is presented in Figures 1 and 2. The methanol extract and petroleum ether fraction at the dose of 400 mg/kg and 200 mg/kg body weight produced highly significant (*P* < 0.0001 and *P* < 0.001) reduction in the number of writhings produced by acetic acid in mice when compared to untreated group. The number of reductions with carbon tetrachloride fraction at 400 mg/kg was also significant (*P* < 0.001). Similarly, petroleum ether fraction at 200 mg/kg and dichloromethane fraction at 400 mg/kg exhibited significant (*P* < 0.05) inhibition. The methanol extract at the dose of 400 mg/kg inhibited writhing by 54% which was comparable to diclofenac sodium of 56%. Among other fractions, petroleum ether fraction at 400 mg/kg produced 49.69% of inhibition, followed by carbon tetrachloride fraction of 40% and 27.27% by dichloromethane fractions at same dose. The percentage inhibition of writhing at the dose of 200 mg/kg for methanol, petroleum ether, and carbon tetrachloride fraction is 45.45%, 39.39%, and 31.51%, respectively, with methanol bearing the highest protection of writhing (Figure 1). The dichloromethane (200 mg/kg) and ethyl acetate fractions at both doses did not show any significant reduction in acetic acid induced writhing.

### 3.3. Radiant Heat Tail Flick Test

**Figure 3:**
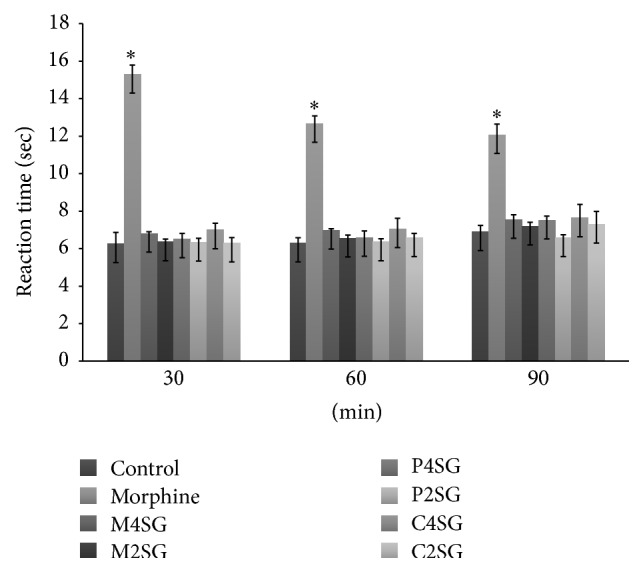
Antinociceptive effect of morphine, M4SG: methanol extract 400 mg/kg, M2SG: methanol extract 200 mg/kg, P4SG: petroleum ether fraction 400 mg/kg, P2SG: petroleum ether 200 mg/kg, C4SG: carbon tetrachloride fraction 400 mg/kg, and C2SG: carbon tetrachloride 200 mg/kg of* S. grossus* in tail flick method. Values are represented in mean ± SEM (*n* = 5). One-way ANNOVA followed by Dunnett's multiple comparisons was performed. *P* < 0.05 was considered significant compared to control, ^*∗*^ *P* < 0.001.

**Figure 4:**
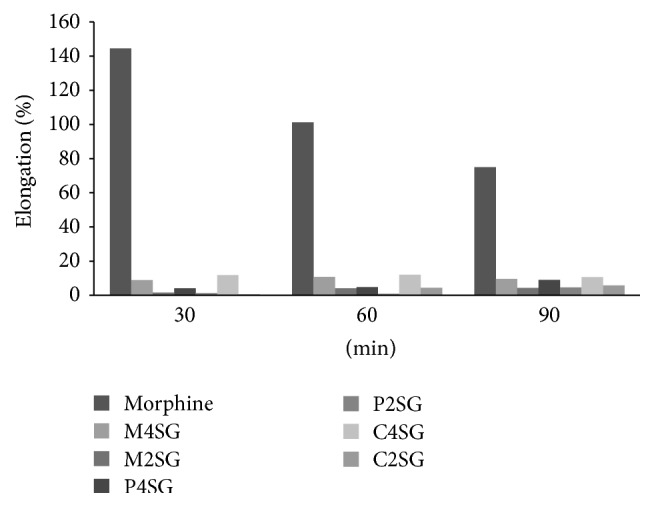
Percent elongation by standard: morphine, M4SG: methanol extract 400 mg/kg, M2SG: methanol extract 200 mg/kg, P4SG: petroleum ether fraction 400 mg/kg, P2SG: petroleum ether 200 mg/kg, C4SG: carbon tetrachloride fraction 400 mg/kg, and C2SG: carbon tetrachloride 200 mg/kg of* S. grossus* in tail flick method.

The result of methanol extract and its different fractions from roots of* Schoenoplectus grossus* when subjected to screening for centrally acting antinociceptive activity on radiant heat tail flick method is tabulated in Figures 3 and 4. The test was performed by taking samples at doses of 200 and 400 mg/kg body weight. The dichloromethane (200 mg/kg) and ethyl acetate fractions at both doses which lacked significant reduction in acetic acid induced writhing were excluded in this test. The untreated animals in control group did not show any significant differences in reaction time during 90-minute period. The standard drug morphine sulphate only produced significant (*P* < 0.0001) increase in reaction time of 15.3 ± 0.495, 12.68 ± 0.399, and 12.08 ± 0.563 in 30 min, 60 min, and 90 min, respectively, while reaction latency time of other Kupchan fractions tested was not found to be significant. The standard drug morphine showed high percentage of elongation by 144.41 at 30 min, 101.27 at 60 min, and 75.07 at 90 min. The percentage elongation of reaction time of carbon tetrachloride fraction and methanol extract at 400 mg/kg body weight was a bit higher among all fractions which were very low compared to standard morphine (Figure 4). No significant results were observed for the treatments except morphine sulphate.

### 3.4. Antipyretic Activity

**Figure 5:**
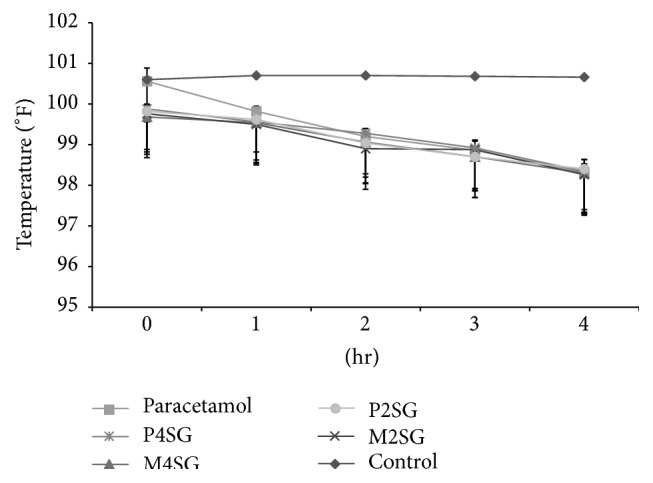
Effect of control, standard: diclofenac sodium, M4SG: methanol extract 400 mg/kg, M2SG: methanol extract 200 mg/kg, P4SG: petroleum ether fraction 400 mg/kg, P2SG: petroleum ether 200 mg/kg, C4SG: carbon tetrachloride fraction 400 mg/kg, and C2SG: carbon tetrachloride 200 mg/kg of* S. grossus* in yeast induced pyrexia in rats.

Antipyretic effects of methanol extract from root of* Schoenoplectus grossus* and its different fractions on rectal temperature are presented in Table 2 and Figure 5. The subcutaneous injection of yeast markedly increased the rectal temperature and the mean increment recorded was 1.24–2°F after 18 hr of administration. The extract and paracetamol treatment groups showed significant effect on rectal temperature (MANOVA, *F* = 2.953, *P* < 0.005, Wilk's Λ = 0.080) with significant reduction of temperature over period of time from 1 hr to 4 hr (MANOVA, *P* < 0.05 and *P* < 0.005). The methanol extract and petroleum ether fractions at the dose of 400 mg/kg and 200 mg/kg body weight significantly attenuated hyperthermia in rats in 1 hr observation (*P* < 0.005) and lowering of temperature was even more significant (*P* < 0.001) from 2 hr to 4 hr observation period in comparison to control. Standard drug paracetamol also significantly inhibited pyrexia (*P* < 0.05 and *P* < 0.001) in early and latter hours of observation time intervals. The different treatment fractions of* S. grossus* and paracetamol lowered the rectal temperature in time dependent manner.

## 4. Discussions

The methanol extract as well as its different fraction from root of* Schoenoplectus grossus* was evaluated for analgesic effect against acetic acid induced visceral pain and the fraction that produced significant activity with this test was further screened for antinociceptive activity using radiant heat tail flick method in mice. The characteristic of pain activity generated by intraperitoneal injection of acetic acid is presented with contraction of abdominal muscle followed by extension of hind limbs and elongation of body part and such constriction is thought to be mediated by local peritoneal receptor [18]. The acetic acid provoked writhing is simple and commonly used method for screening analgesic drugs [19]. The administration of acetic acid is responsible for the release of endogenous substances which are supposed to excite the nerve endings causing the pain [19, 20]. Studies have revealed the accumulation of higher levels of prostaglandins especially PGE2, PGF2*α* [21], PGI2 [22], lipoxygenase products [23], and peritoneal mast cells [24], in peritoneal fluids treated with algogenic acetic acid. Acetic acid further potentiates the pain through capillary permeability [25].

The major contribution of prostaglandins to eliciting pain response is mainly due to interaction with endogenous mediators like histamine, serotonin, bradykinin, and substance P which further stimulate the sensitization of pain receptors to these mediators [26]. It is well established that NSAIDs relieve the pain response peripherally by inhibiting production of prostaglandins, thromboxane, and other inflammatory mediators by acting on cyclooxygenase enzymes. Any substance lessening the number of constrictions induced by acetic acid can be considered to have analgesic potential. The methanol extract of* S. grossus* and its fraction significantly reduced the number of writhings in dose dependent manner. This strongly suggests that the plant under study possesses peripheral analgesic property, possibly mediated through same mechanism of inhibition of prostaglandins generating pathways and local peritoneal inflammation.

The centrally mediated antinociceptive activity was evaluated using tail flick method [27], which measures the time taken to withdraw tail from the thermal source, recorded as reaction time and opioid-like analgesics increase threshold time of such tail flicking [28]. The behavior of pain resulting from this method is based on reflex mediated at spinal level which is considered more sensitive to other thermal nociceptive models like hot plate method [29, 30]. This method has also advantage of differentiating central opioid-like analgesics from peripheral analgesics [31]. In our study, the methanol extract and its fraction did not significantly prolong reaction time of tail flicking during all observations indicating that the plant extract might not be acting spinally except morphine. These findings from the study suggest that plant is devoid of central antinociceptive activity and observed analgesic effect is supposed to be mediated peripherally. The antinociceptive study performed on* Acanthus montanus* revealed similar type of result with positive effect on acetic acid induced method but lacking activity in tail flick method supports our findings [32].

Yeast induced fever, which represents pathogenic fever, presents an economical and reliable method for assessing new antipyretics [16]. The presence of proteins in yeast is linked to fever via inflammatory reaction in this method [33]. Further, the production of proinflammatory cytokines such as interleukin-1*β* (IL-1*β*) and IL-6, interferon-*α* (IFN-*α*), and tumor necrosis factor-*α* (TNF-*α*) and prostaglandins like PGE2 and PGI2 are responsible for elevating the body temperature by acting on brain [34–36]. Antipyretics such as paracetamol used in management of fever act through several ways by reducing levels of prostaglandins acting on cyclooxygenase enzymes, enhancing antipyretic message within brain and stimulating anti-inflammatory signals at injury site [37]. The methanol and petroleum ether fractions of* S. grossus* significantly lowered the temperature in yeast induced pyrexia. The lowering of temperature was almost in a similar manner to that of reference drug, paracetamol, suggesting that the plant have antipyretic property which can be assumed to be mediated through interference of prostaglandin synthesis and inhibition of cytokines release. Of the portioned fractions, the methanol extracts and petroleum ether fractions producing significant effect on acetic acid induced writhing were only included in evaluation of antipyretic activity while carbon tetrachloride, dichloromethane, and ethyl acetate fractions were excluded. The studies conducted by various researchers have revealed that the medicinal plants showing analgesic property have also demonstrated antipyretic as well as anti-inflammatory activities [38, 39] as the mechanism for suppression of pain, fever, and inflammation can be correlated via inhibition of inflammatory mediators. This further reinforces the evaluation of root extract of this plant for anti-inflammatory potential.

Several studies conducted on medicinal plants revealing the presence of secondary metabolites like flavonoids and alkaloids have been linked to possess analgesic, antipyretic, and other properties [38, 40]. There are also similar studies on medicinal plants suggesting role of tannins in suppression of pain and inflammatory activities [31, 41]. Flavonoids are the main constituents that have capacity to interfere with eicosanoids biosynthesis pathways [42–44] and are suggested to decrease the release of arachidonic acid through inhibition of neutrophils degranulation [45]. Both of these actions result in suppression of inflammatory mediators like prostaglandins and lipoxygenase end products responsible for inflammation, pain, and fever. The above-mentioned phytochemical constituents have been confirmed in preliminary phytochemical screening of* S. grossus*. Thus, both analgesic and antipyretic activities observed in this study can be assumed due to presence of one or several phytoconstituents detected in the plant. The various components of each extract and their bioactive compounds were not separated which remains the limitations in study.

## 5. Conclusion

The methanol extract and its different fractions from root of* S. grossus* displayed significant peripheral analgesic potential and antipyretic property. The central antinociceptive activity was absent. Since this is a pioneer work further studies are necessary to validate this result and other detailed studies on compound identification and isolation and underlying mechanism for the observed effect are essential to guarantee its clinical use.

## Figures and Tables

**Table 1:** Phytochemical constituents in root extract of *S. grossus* and its fractions.

Phytochemical	Methanol	Petroleum ether	Carbon tetrachloride	Dichloromethane	Ethyl acetate
Alkaloids	+	+	+	+	−
Flavonoids	+	+	+	+	+
Tannins	+	+	−	−	+
Steroids	−	−	−	−	−

“+” = presence; “−” = absence.

**Table 2:** Antipyretic effect of methanol extract from root of *S. grossus* and its fraction in yeast induced pyrexia in rats.

Treatment	Dose (mg/kg)	Basal temp. °F	Rectal temperature (°F)				
			0 hour (after 18 hr)	1 hr	2 hr	3 hr	4 hr
Control		99.0	100.6 ± 0.300	100.7 ± 0.277	100.7 ± 0.187	100.68 ± 0.191	100.66 ± 0.194
Paracetamol	100	98.56	100.56 ± 0.326	99.82 ± 0.124^*∗∗∗*^	99.2 ± 0.192^*∗*^	98.86 ± 0.051^*∗*^	98.32 ± 0.097^*∗*^
Methanol	400	98.42	99.68 ± 0.193^*∗∗∗*^	99.54 ± 0.229^*∗∗*^	99.06 ± 0.266^*∗*^	98.70 ± 0.210^*∗*^	98.3 ± 0.214^*∗*^
Methanol	200	98.52	99.76 ± 0.229	99.5 ± 0.207^*∗∗*^	98.9 ± 0.217^*∗*^	98.88 ± 0.206^*∗*^	98.26 ± 0.378^*∗∗*^
Petroleum ether	400	98.54	99.88 ± 0.097	99.56 ± 0.117^*∗∗*^	99.28 ± 0.116^*∗*^	98.92 ± 0.193^*∗*^	98.34 ± 0.189^*∗*^
Petroleum ether	200	98.48	99.82 ± 0.169	99.62 ± 0.188^*∗∗*^	99.04 ± 0.250^*∗*^	98.7 ± 0.263^*∗*^	98.40 ± 0.230^*∗*^

Values are represented in mean ± SEM (*n* = 5). MANNOVA followed by Dunnett's multiple comparisons was performed to analyze this dataset. *P* < 0.05 was considered significant compared to control; ^*∗*^ *P* < 0.001, ^*∗∗*^ *P* < 0.005, and ^*∗∗∗*^ *P* < 0.05.

## References

[1] Castellsague J., Riera-Guardia N., Calingaert B. (2012). Individual NSAIDs and upper gastrointestinal complications: a systematic review and meta-analysis of observational studies (the SOS Project). *Drug Safety*.

[2] Ofman J. J., MacLean C. H., Straus W. L. (2002). A metaanalysis of severe upper gastrointestinal complications of nonsteroidal antiinflammatory drugs. *The Journal of Rheumatology*.

[3] Hippisley Cox J., Coupland C. (2005). Risk of myocardial infarction in patients taking cyclo-oxygenase-2 inhibitors or conventional non-steroidal anti-inflammatory drugs: population based nested case-control analysis. *British Medical Journal*.

[4] Jüni P., Nartey L., Reichenbach S., Sterchi R., Dieppe P. A., Egger P. M. (2004). Risk of cardiovascular events and rofecoxib: cumulative meta-analysis. *The Lancet*.

[5] Mamdani M., Juurlink D. N., Lee D. S. (2004). Cyclo-oxygenase-2 inhibitors versus non-selective non-steroidal anti-inflammatory drugs and congestive heart failure outcomes in elderly patients: a population-based cohort study. *The Lancet*.

[6] Lenzer J. (2005). FDA advisers warn: COX 2 inhibitors increase risk of heart attack and stroke. *British Medical Journal*.

[7] Benyamin R., Trescot A. M., Datta S. (2008). Opioid complications and side effects. *Pain Physician*.

[8] Phillips C. J. (2006). Economic burden of chronic pain. *Expert Review of Pharmacoeconomics & Outcomes Research*.

[9] Hewitt D. J., Hargreaves R. J., Curtis S. P., Michelson D. (2009). Challenges in analgesic drug development. *Clinical Pharmacology & Therapeutics*.

[10] Robinon M. R., Zhang X. (2011). *The World Medicine Situation (Traditional Medicines: Global Situation, Issues and Challenges). Geneva*.

[11] Ghani A. (2003). *Medicinal Plants of Bangladesh*.

[12] Beckett A. H., Stenlake J. B. (1986). *Chromatography: Practical Pharmaceutical Chemistry*.

[13] Trease G. E., Evans W. (1989). *A Text Book of Pharmacognosy*.

[14] Koster R., Anderson M., De Beer E. J. (1959). Acetic acid-induced analgesic screening. *Federation Proceedings*.

[15] D'amour F. E., Smith D. L. (1941). A method for determining loss of pain sensation. *Journal of Pharmacology and Experimental Therapeutics*.

[16] Tomazetti J., Ávila D. S., Ferreira A. P. (2005). Baker yeast-induced fever in young rats: characterization and validation of an animal model for antipyretics screening. *Journal of Neuroscience Methods*.

[17] Turner R. A. (1965). *Screening Methods in Pharmacology*.

[18] Bentley G. A., Newton S. H., Starr J. (1983). Studies on the antinociceptive action of *α*-agonist drugs and their interactions with opioid mechanisms. *British Journal of Pharmacology*.

[19] Collier H. O., Dinneen L. C., Johnson C. A., Schneider C. (1968). The abdominal constriction response and its suppression by analgesic drugs in the mouse. *British Journal of Pharmacology and Chemotherapy*.

[20] Pavao-de-Souza G. F., Zarpelon A. C., Tedeschi G. C. (2012). Acetic acid- and phenyl-p-benzoquinone-induced overt pain-like behavior depends on spinal activation of MAP kinases, PI_3_K and microglia in mice. *Pharmacology Biochemistry and Behavior*.

[21] Deraedt R., Jouquey S., Delevallée F., Flahaut M. (1980). Release of prostaglandins E and F in an algogenic reaction and its inhibition. *European Journal of Pharmacology*.

[22] Berkenkopf J. W., Weichman B. M. (1988). Production of prostacyclin in mice following intraperitoneal injection of acetic acid, phenylbenzoquinone and zymosan: its role in the writhing response. *Prostaglandins*.

[23] Levine J. D., Lau W., Kwiat G., Goetzl E. J. (1984). Leukotriene B4 produces hyperalgesia that is dependent on polymorphonuclear leukocytes. *Science*.

[24] Ribeiro R. A., Vale M. L., Thomazzi S. M. (2000). Involvement of resident macrophages and mast cells in the writhing nociceptive response induced by zymosan and acetic acid in mice. *European Journal of Pharmacology*.

[25] Amico-Roxas M., Caruso A., Trombadore S., Scifo R., Scapagnini U. (1984). Gangliosides antinociceptive effects in rodents. *Archives Internationales de Pharmacodynamie et de Therapie*.

[26] Davies P., Bailey P. J., Goldenberg M. M., Ford-Hutchinson A. W. (1984). The role of arachidonic acid oxygenation products in pain and inflammation. *Annual Review of Immunology*.

[27] Vogel H. (2007). *Drug Discovery and Evaluation: Pharmacological Assays*.

[28] Tjølsen A., Hole K., Gebhart G., Schmidt R. (2013). Tail-Flick test. *Encyclopedia of Pain*.

[29] Chapman C. R., Casey K. L., Dubner R., Foley K. M., Gracely R. H., Reading A. E. (1985). Pain measurement: an overview. *Pain*.

[30] Gårdmark M., Höglund A. U., Hammarlund-Udenaes M. (1998). Aspects on tail-flick, hot-plate and electrical stimulation tests for morphine antinociception. *Pharmacology and Toxicology*.

[31] Fan S., Ali N. A., Basri D. F. (2014). Evaluation of analgesic activity of the methanol extract from the galls of *Quercus infectoria* (Olivier) in rats. *Evidence-Based Complementary and Alternative Medicine*.

[32] Asongalem E. A., Foyet H. S., Ekobo S., Dimo T., Kamtchouing P. (2004). Antiinflammatory, lack of central analgesia and antipyretic properties of *Acanthus montanus* (*Ness*) *T. Anderson*. *Journal of Ethnopharmacology*.

[33] Pasin J. S. M., Ferreira A. P. O., Saraiva A. L. L. (2010). Diacerein decreases TNF-*α* and IL-1*β* levels in peritoneal fluid and prevents Baker's yeast-induced fever in young rats. *Inflammation Research*.

[34] Luheshi G. N. (1998). Cytokines and fever: mechanisms and sites of action. *Annals of the New York Academy of Sciences*.

[35] Saper C. B., Breder C. D. (1994). The neurologic basis of fever. *The New England Journal of Medicine*.

[36] Veale W. L., Cooper K. E., Pittman Q. J., Ramwell P. (1977). Role of prostaglandins in fever and temperature regulation. *The Prostaglandins*.

[37] Aronoff D. M., Neilson E. G. (2001). Antipyretics: mechanisms of action and clinical use in fever suppression. *The American Journal of Medicine*.

[38] Afsar T., Khan M. R., Razak S., Ullah S., Mirza B. (2015). Antipyretic, anti-inflammatory and analgesic activity of *Acacia hydaspica* R. Parker and its phytochemical analysis. *BMC Complementary and Alternative Medicine*.

[39] Rauf A., Uddin G., Siddiqui B. S., Muhammad N., Khan H. (2014). Antipyretic and antinociceptive activity of *Diospyros lotus* L. in animals. *Asian Pacific Journal of Tropical Biomedicine*.

[40] Kumar A., Agarwal K., Maurya A. K. (2015). Pharmacological and phytochemical evaluation of *Ocimum sanctum* root extracts for its antiinflammatory, analgesic and antipyretic activities. *Pharmacognosy Magazine*.

[41] Viana G. S. B., Bandeira M. A. M., Moura L. C., Souza-Filho M. V. P., Matos F. J. A., Ribeiro R. A. (1997). Analgesic and antiinflammatory effects of the tannin fraction from *Myracrodruon urundeuva* Fr. All. *Phytotherapy Research*.

[42] Goda Y., Kiuchi F., Shibuya M., Sankawa U. (1992). Inhibitors of prostaglandin biosynthesis from *Dalbergia odorifera*. *Chemical and Pharmaceutical Bulletin*.

[43] Dames J., Bourdon V., Remacle-Uolon G., Lecomte J. (1985). Pro-inflammatory flavonoids which are inhibitors of prostaglandin biosynthesis. *Prostaglandins, Leukotrienes and Medicine*.

[44] Robak J., Gryglewski R. J. (1996). Bioactivity of flavonoids. *Polish Journal of Pharmacology*.

[45] Tordera M., Ferrandiz M. L., Alcaraz M. J. (1994). Influence of anti-inflammatory flavonoids on degranulation and arachidonic acid release in rat neutrophils. *Zeitschrift für Naturforschung C—Journal of Biosciences*.

